# Prospective, Multicentre, Nationwide Clinical Data from 600 Cases of Acute Pancreatitis

**DOI:** 10.1371/journal.pone.0165309

**Published:** 2016-10-31

**Authors:** Andrea Párniczky, Balázs Kui, Andrea Szentesi, Anita Balázs, Ákos Szűcs, Dóra Mosztbacher, József Czimmer, Patrícia Sarlós, Judit Bajor, Szilárd Gódi, Áron Vincze, Anita Illés, Imre Szabó, Gabriella Pár, Tamás Takács, László Czakó, Zoltán Szepes, Zoltán Rakonczay, Ferenc Izbéki, Judit Gervain, Adrienn Halász, János Novák, Stefan Crai, István Hritz, Csaba Góg, János Sümegi, Petra Golovics, Márta Varga, Barnabás Bod, József Hamvas, Mónika Varga-Müller, Zsuzsanna Papp, Miklós Sahin-Tóth, Péter Hegyi, on behalf of the Hungarian Pancreatic Study Group

**Affiliations:** 1 Heim Pál Children's Hospital, Budapest, Hungary; 2 First Department of Medicine, University of Szeged, Szeged, Hungary; 3 Institute for Translational Medicine, University of Pécs, Pécs, Hungary; 4 First Department of Surgery, Semmelweis University, Budapest, Hungary; 5 Department of Pediatrics, Balassa János Hospital of County Tolna, Szekszárd, Hungary; 6 First Department of Medicine, University of Pécs, Pécs, Hungary; 7 Szent György University Teaching Hospital of County Fejér, Székesfehérvár, Hungary; 8 Pándy Kálmán Hospital of County Békés, Gyula, Hungary; 9 Bács-Kiskun County University Teaching Hospital, Kecskemét, Hungary; 10 Healthcare Center of County Csongrád, Makó, Hungary; 11 Borsod-Abaúj-Zemplén County Hospital and University Teaching Hospital, Miskolc, Hungary; 12 First Department of Medicine, Semmelweis University, Budapest, Hungary; 13 Dr. Réthy Pál Hospital, Békéscsaba, Hungary; 14 Dr. Bugyi István Hospital, Szentes, Hungary; 15 Bajcsy-Zsilinszky Hospital, Budapest, Hungary; 16 Department of Molecular and Cell Biology, Boston University Henry M. Goldman School of Dental Medicine, Boston, United States of America; 17 Hungarian Academy of Sciences – University of Szeged, Momentum Gastroenterology Multidisciplinary Research Group, Szeged, Hungary; Klinikum rechts der Isar der Technischen Universitat München, GERMANY

## Abstract

**Objective:**

The aim of this study was to analyse the clinical characteristics of acute pancreatitis (AP) in a prospectively collected, large, multicentre cohort and to validate the major recommendations in the IAP/APA evidence-based guidelines for the management of AP.

**Design:**

Eighty-six different clinical parameters were collected using an electronic clinical research form designed by the Hungarian Pancreatic Study Group.

**Patients:**

600 adult patients diagnosed with AP were prospectively enrolled from 17 Hungarian centres over a two-year period from 1 January 2013.

**Main Results:**

With respect to aetiology, biliary and alcoholic pancreatitis represented the two most common forms of AP. The prevalence of biliary AP was higher in women, whereas alcoholic AP was more common in men. Hyperlipidaemia was a risk factor for severity, lack of serum enzyme elevation posed a risk for severe AP, and lack of abdominal pain at admission demonstrated a risk for mortality. Abdominal tenderness developed in all the patients with severe AP, while lack of abdominal tenderness was a favourable sign for mortality. Importantly, lung injury at admission was associated with mortality. With regard to laboratory parameters, white blood cell count and CRP were the two most sensitive indicators for severe AP. The most common local complication was peripancreatic fluid, whereas the most common distant organ failure in severe AP was lung injury. Deviation from the recommendations in the IAP/APA evidence-based guidelines on fluid replacement, enteral nutrition and timing of interventions increased severity and mortality.

**Conclusions:**

Analysis of a large, nationwide, prospective cohort of AP cases allowed for the identification of important determinants of severity and mortality. Evidence-based guidelines should be observed rigorously to improve outcomes in AP.

## Introduction

Acute pancreatitis (AP) is a serious disease with high mortality [1]. The reported incidence is variable in different countries (10–100/100,000 people [2], and AP is a leading cause of acute hospitalization for gastrointestinal disorders [3]. Published studies on the clinical characteristics of AP [4, 5] have mostly been based on retrospective cohorts or prospectively collected data from 200–300 cases [4, 5]. Large, nationwide, prospectively collected cohorts are needed. Adherence to treatment guidelines has been documented to reduce mortality and/or severity of AP [1]. Therefore, dissemination and validation of newly described guidelines are important. The IAP/APA guidelines were approved by 171 experts worldwide [6]; however, the recommendations have not yet been validated in large prospective cohorts. Therefore, the main goals of our study were to (1) analyse the course of AP in a prospectively collected cohort of patients from Hungarian centres and (2) to validate the major recommendations in the IAP/APA evidence-based guidelines for the management of AP.

## Methods

The study was approved by the Scientific and Research Ethics Committee of the Medical Research Council (22254-1/2012/EKU). All the participants provided written informed consent to participate in this study. The ethics committee have carefully checked and approved the consent procedure. The Hungarian Pancreatic Study Group (HPSG) was established in 2011 to improve patient care for pancreatic diseases [7–11]. To achieve our aims, we developed a uniform prospective electronic data registry (www.pancreas.hu), which formed the base for our data collection. For this HPSG study cohort, 600 patients in Hungary were prospectively enrolled for two years between 1 January 2013 and 1 January 2015. Centre distribution is indicated in S1 Fig. Diagnosis of AP was based on recommendation A1 in the IAP/APA guidelines [6]. Two of the following alterations were confirmed in each patient: abdominal pain (clinical symptom), pancreatic enzyme elevation at least three times above the upper limit and morphological changes (imaging changes). Eighty-six different parameters were collected (see S2 Fig). Overall, 77% of the requested data were provided by investigators. The missing data were either not measured (e.g. breath rate at admission) or not investigated (e.g. procalcitonin levels at admission). Only four of the collected parameters were not analysed due to a high amount of missing data.

Please note that some of the presented data are difficult to measure (for example alcohol consumption, pain, type of pain, tenderness etc.) and have potential risk for bias, therefore these data need to be interpreted with caution.

### Statistical Analyses

A biostatistics consultancy (AdWare Research Ltd., Balatonfüred, Hungary) aided us in selecting and using the adequate methods for the statistical analyses. For descriptive statistics, the number of patients, mean, standard deviation (SD), minimum, median and maximum values were calculated for continuous variables, and the case number and percentage were computed for categorical values. For inferential statistics, the following statistical tests were used for determining statistical significance of differences between groups. To compare two groups of independent samples, the t-test was applied for normally distributed data and the Mann–Whitney U test for non-normal data. To compare more than two groups, one-way ANOVA with the Bonferroni adjustment method was used for normally distributed data with homogenous group-wise standard deviation; Brown-Forsythe Levene-type test was applied to test of variance homogeneity; the Welch test followed by the Games–Howell post hoc test for normally distributed data with heterogeneous group-wise standard deviation; and the Kruskal–Wallis test followed by the Bonferroni p value adjustment method for non-normal data. The association between categorical variables was examined with the Chi-square test and Fisher's exact test. For categorical variables the multiple comparison between groups were not applied. The relevant statistical tests are also described in the legends to the figures. Statistical analyses were prepared by SPSS 19.0.0. Brown-Forsyte Levene test was prepared by R Studio Version 0.99.896-2009-2016 R studio, Inc., Lawstat package.

## Results

### Epidemiology and Aetiology

**Fig 1:**
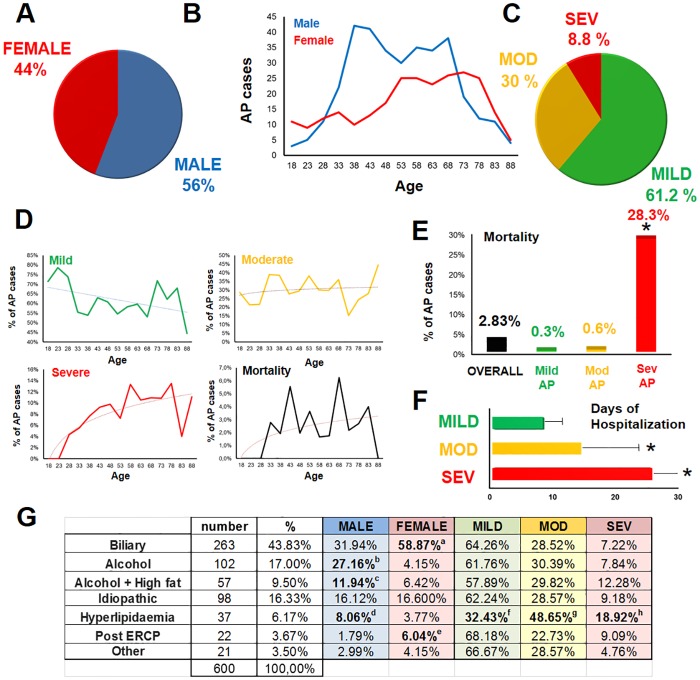
Epidemiology and aetiology. **A**. Sex distribution of AP cases. **B.** Age distribution of AP cases. **C.** AP severity groups. Mod: moderate; sev: severe. **D.** Age distribution of mild, moderate and severe AP cases and mortality. **E.** Overall mortality and distribution in the severity groups. p<0.001 was between the severe and other groups according to Fisher’s exact test. **F.** Days of hospitalization. Mann-Whitney U test with Bonferroni correction was used to compare the group pairs (p<0.001 between groups). **G.** Aetiology of AP. (**a:** p<0.001; **b:** p<0.001; **c:** p = 0.022, **d:** p = 0.030; **e:** p = 0.006; **f:** p<0.001; **g:** p = 0.011; **h:** p = 0.025).

In our cohort, 56% (n = 335) of the patients were male, and 44% (n = 265) were female (Fig 1A). With regard to the age distribution of the cases, AP incidence in the males increased between 33 and 38 years and remained high until 68 years, after which it sharply declined. In the females, the highest incidence was between 53 and 78 years (Fig 1B). The majority (61.2%) of the cases were mild, 30% were moderate, and 8.8% were severe, according to the revised Atlanta classification [12] (Fig 1C). The incidence of severe AP demonstrated an age-dependent rise between 23 and 58 years. In contrast, the frequency of mild or moderate AP did not show a similar age distribution (Fig 1D), suggesting that age may be a risk factor for disease severity.

Overall mortality was 2.83% (17 deaths/600 patients) in the cohort. Mortality was higher in severe AP (28.3%; p<0.001) versus moderate (0.6%) and mild AP (0.3%) (Fig 1E). Analyses of age distribution for mortality revealed two peaks at 43 and 68 years (Fig 1D). There was no relevant difference in mortality between mild and moderate AP. The length of hospitalization showed significant differences between these groups (mild: 8.3±0.2 days; moderate: 14.6±0.5 days; severe: 26.2±3.1 days; p<0.001) (Fig 1F). Mortality peaked between days 1 and 4 (early mortality) and days 11 and 14 (late mortality) (S3 Fig). The cost of treatment (calculating only the costs of medications, examinations and interventions) for mild AP was HUF 99,006 (approximately 330 euros); however, it increased to HUF 1,725,135 (approximately 5,750 euros) for severe AP, based on an average of 10 patients per group.

The most common aetiology of AP was biliary disease (43.8%) and alcohol abuse (26.5%) (Fig 1G). In females, the frequency of biliary AP was almost twice as high as in males (58.9% vs. 31.9%). In contrast, alcoholic AP was almost four times more common in males (39.1%) versus females (10.6%). When alcohol plus high-fat diet was considered as a separate etiological group, the frequency was still higher among males (11.9%) versus females (6.4%). Hyperlipidaemia was more frequently observed in males (8.1%) versus females (3.8%). Relative to other aetiologies, the frequency of hyperlipidaemia was significantly lower in mild AP, whereas it was higher in moderate and severe AP, indicating that hyperlipidaemia is a risk factor for severity. The number of idiopathic cases was comparable in males (16.1%) and females (16.6%) (Fig 1G). Post-ERCP AP was significantly more prevalent in females (6%) versus males (1.8%). Although it is true that more female patients have ERCP than male patients (the difference is less than 3.3%) but this rather reflects the risk difference. Notably, female gender is an independent risk factor for post-ERCP AP (ESGE Guideline 2014).

**Fig 2:**
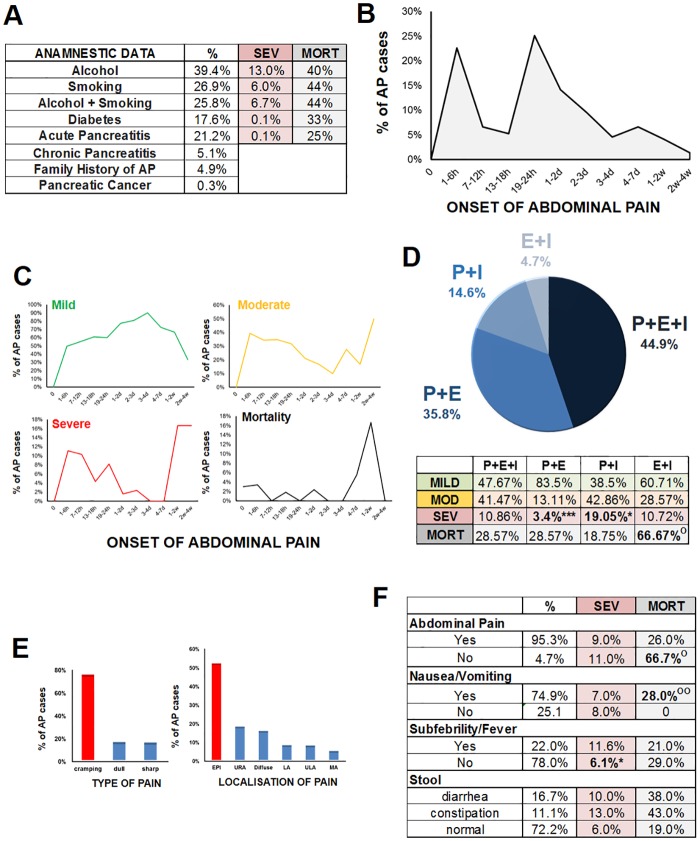
Diagnosis, anamnestic data and symptoms at admission. **A.** Anamnestic data. The percentages of severe AP and mortality in severe AP are also shown in relation to alcohol consumption, smoking, diabetes and history of earlier AP. **B.** Relationship between time of onset of abdominal pain and presentation at ER units. **C.** Time of onset of abdominal pain and presentation at ER in the three severity groups and association with mortality in the severe group. **D.** Diagnosis. Distribution of diagnostic criteria in the overall cohort (pie chart) and in the three severity groups (table) and association with mortality in severe AP (table). P: pain; E: enzyme elevation; I: imaging alteration. ^O^ p = 0.189 (Fisher’s exact test) * p = 0.005 (Chi-square test) *** p<0.001 (Chi-square test). **E.** Type and localisation of abdominal pain. EPI: epigastric pain; URA: upper right abdomen; ULA: upper left abdomen; MD: middle abdomen; L: lower abdomen; D: diffuse. **F.** Symptoms in the entire cohort and in the severe AP group and association with mortality in the severe AP group. ^O^ p = 0.189 (Fisher’s exact test) ^OO^ p = 0.051 (Chi-square test) * p = 0.029 (Chi-square test).

Anamnestic data collected at admission revealed that 21.2% of AP was recurrent (Fig 2A) and 4.9% had a family history of AP. Neither mortality nor severity was affected by recurrence. History of alcohol consumption or smoking was associated with higher mortality in severe AP; however, this difference did not reach statistical significance due to the small number of such cases. The combined presence of both toxic factors did not raise mortality further (Fig 2A).

### Diagnosis, Anamnestic Data and Symptoms at Admission

The majority of AP patients usually presented at emergency departments 1–6 hours or 19–24 hours after the onset of abdominal pain (Fig 2B). Not seeking medical attention during the first four days of AP strongly heightened the risk for severe AP and mortality (Fig 2C).

Diagnosis of AP was based on the two-thirds rule as described in *Methods*. The large majority (95.3%) of the patients suffered from abdominal pain, 85.4% experienced serum pancreatic enzyme elevation, and 64.2% had imaging alterations (oedema or peripancreatic fluid) (Fig 2D). In 44.9% of the cases, all three diagnostic criteria were present. In 80.7% of the cases, diagnosis of AP could be established on the basis of abdominal pain and a rise in pancreatic enzyme. Importantly, the lack of an increase in enzyme was a risk factor for severe pancreatitis, whereas the lack of abdominal pain demonstrated a risk for mortality. The absence of imaging alterations in the pancreas significantly decreased the risk of severe AP (Fig 2D).

The pain was mostly epigastric in origin as well as from cramping (Fig 2E). In addition to abdominal pain, nausea and/or vomiting were the other most frequent complaints (74.9%) (Fig 2F). Importantly, mortality was not observed among patients without nausea and/or vomiting as AP symptoms. Other clinical symptoms and their relation with severity and mortality are described in Fig 2F.

## Physical Examination at Admission

**Fig 3:**
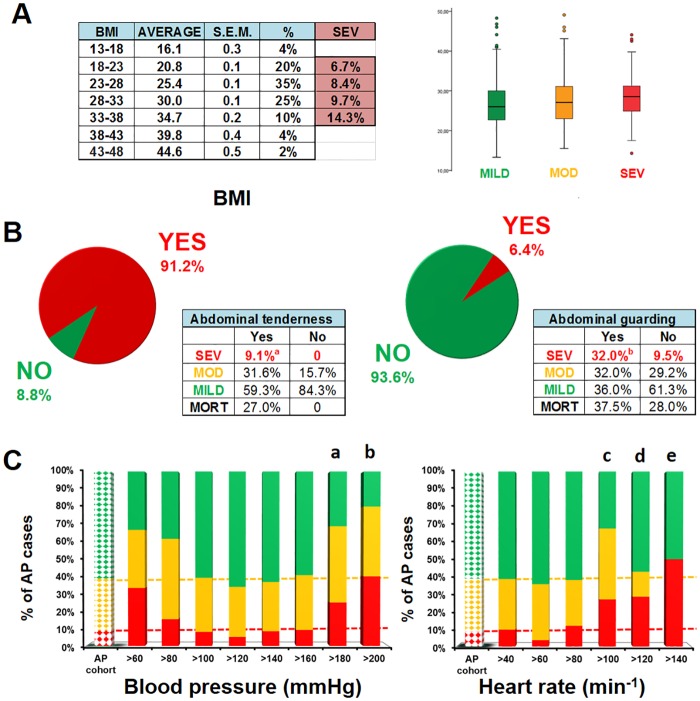
Physical examination at admission. **A.** Body Mass Index (BMI) in the three AP severity groups. BMI values for 90% of the cohort were between 18 and 38. Although the tendency suggests that a rise in BMI increases the risk for severe AP, statistical analyses showed no significant differences between the groups. **B.** Abdominal tenderness and guarding in the three AP severity groups. Both abdominal tenderness and guarding were more frequent in severe AP (**a:** p = 0.025; **b:** p<0.001; Chi-square test). Mortality in the severe AP group is shown. **C.** Systolic blood pressure and heart rate in the three AP severity groups. The first dotted column represents the entire cohort. Green: mild AP; yellow: moderate AP; red: severe AP. (**a:** p = 0.027; **b:** p = 0.016; **c:** p<0.001; **d:** p = 0.071; **e:** p = 0.042; Chi-square test).

Although a tendency for association was observed between the rise in BMI and severity of AP, a significant difference was not found when patients with different BMI levels were compared for severity or when the three severity groups were compared with respect to BMI averages (Fig 3A). With regard to the physical examination of the abdomen, tenderness was detected in 91.2% of the cases (Fig 3B). Importantly, abdominal tenderness developed in all the patients suffering from severe AP; however, the lack of this symptom was favourable for mortality. Abdominal guarding developed in 6.4% of the patients during AP, and this symptom was associated with increased mortality in severe AP (Fig 3B). Systolic blood pressure above 180 Hgmm or elevation of heart rate above 100 was significantly connected with severe AP (Fig 3C).

There was a lack of measurement of breath rates among doctors regarding the possibility of lung injury. The number of documented respiratory rate measurements at admission was only 2.5%.

### Imaging at Admission

All the patients had either abdominal US or CT at admission. During abdominal US, only 74.3% of the investigators described the status of the lungs (S4 Fig). Thoracic X-ray was performed in 37.7% of the cases and thoracic CT was conducted in 6.5% of them. Pleural fluid and/or pulmonary infiltrates were found in 6.7% of the US examinations, 26.6% of the X-rays and 75.6% of the CT scans. The distribution of positive findings among the different imaging modalities indicates that doctors are more likely to order chest X-rays or CTs when the clinical picture suggests moderate or severe AP. Importantly, mortality was not observed in the absence of lung injury at admission (S4 Fig).

### Laboratory Parameters at Admission

**Fig 4:**
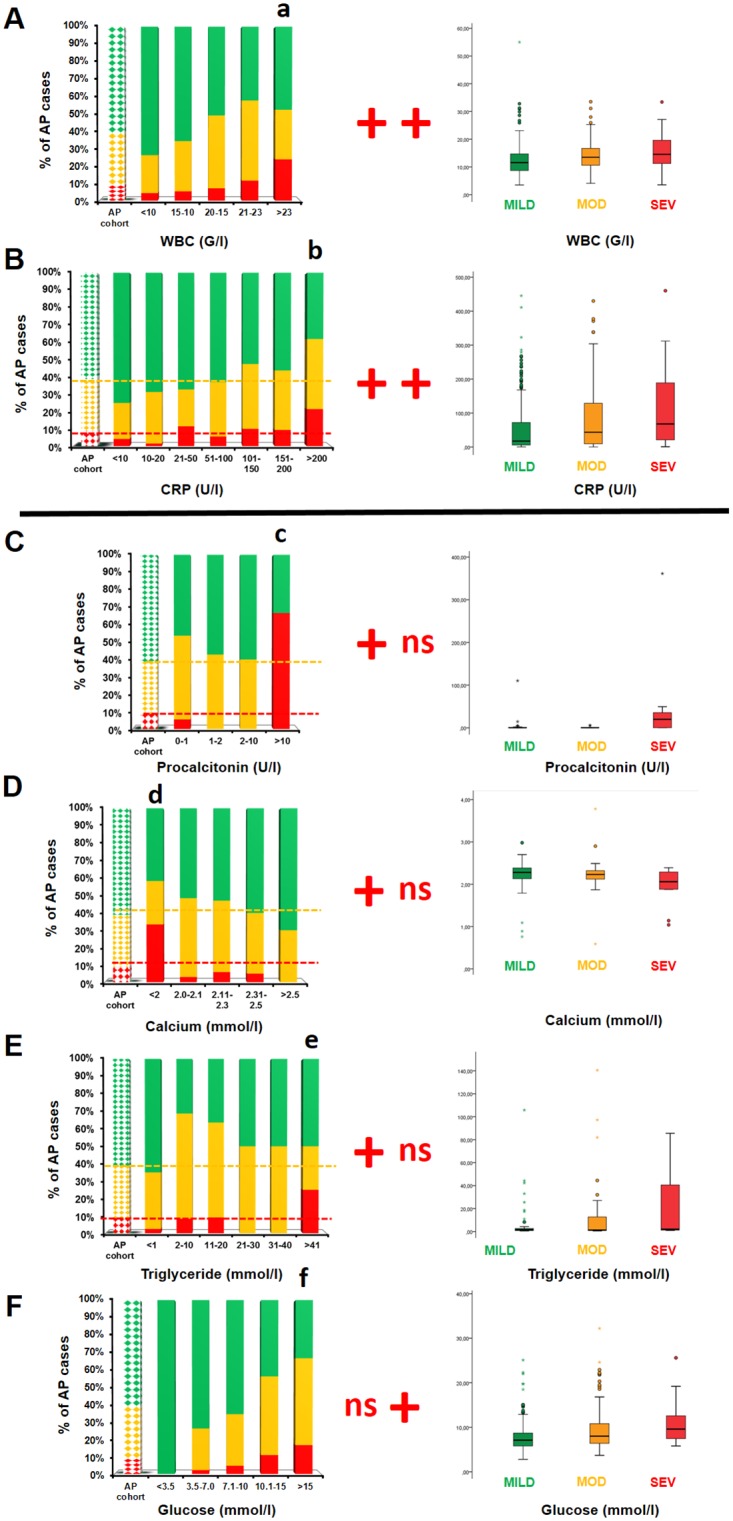
Laboratory parameters in AP. The only parameters shown are where statistical differences were found between the AP severity groups. Green: mild AP; yellow: moderate AP; red: severe AP; **ns:** no significant difference (p>0.05); **+:** significant difference (p<0.05). In the left-hand panel of graphs, laboratory parameters were analysed by distinct values, grouped in ranges. The first dotted column represents the AP severity groups of the entire cohort. Here, the Chi-square test was employed. In the right-hand panel of graphs, the average laboratory parameters were compared in the three AP severity groups. Here, we used the Kruskal–Wallis test and Mann–Whitney U test with a Bonferroni correction to compare the pairs of groups under examination. **A.** White blood cell count (WBC, n = 21–204). A WBC count above 23,000/μL was associated with elevated risk of severe AP (**a:** p = 0.020), and the average WBC counts also showed significant differences between the mild versus moderate and mild versus severe AP groups (p<0.001). **B.** C-reactive protein (CRP: n = 32–144). CRP above 200 mg/L was associated with severe AP (**b:** p = 0.007). In addition, average CRP levels differed significantly between the mild versus moderate and mild versus severe AP groups (p<0.001). **C.** Procalcitonin (PCT, n = 5–54). PCT levels above 10 U/L were associated with elevated risk of severe AP (**c:** p<0.001); however, average PCT levels did not differ significantly between the three AP severity groups (p = 0.143). **D.** Calcium (Ca, n = 12–40). Ca levels below 2 mmol/L were associated with a heightened risk of severe AP (**d:** p = 0.004); however, the average calcium levels did not differ significantly between the three AP severity groups (p = 0.077). **E.** Triglycerides (Tg: n = 10–48). Tg levels above 41 mmol/L were associated with greater risk of severe AP (**e:** p = 0.012); however, average Tg levels did not differ significantly between the three AP severity groups (p = 0.153). **F.** Glucose. (n = 3–175). Significant differences in severity associated with particular glucose levels were not found (**f:** p = 0.191); however, average glucose levels differed significantly between the mild versus moderate and mild versus severe AP groups (p<0.001).

Laboratory parameters were evaluated using two methods. The distribution of distinct values (grouped in ranges) was calculated within the three AP severity groups (Fig 4, left panels), and average values were compared between the three severity groups (Fig 4, right panels).

A white blood cell (WBC) count above 23,000/μL was associated with severe AP (OR 3.2; 95% CI 1.1–9.2). Furthermore, the average WBC counts differed significantly between the mild vs. moderate and the mild vs. severe AP groups (Fig 4A).

The level of C-reactive protein (CRP) above 200 mg/L was associated with severe AP (OR 2.8; 95% CI 1.3–6.2). The average CRP levels differed significantly between the mild vs. moderate and the mild vs. severe AP groups (Fig 4B).

Procalcitonin (PCT) levels above 10 U/L were associated with severe AP (OR 20.6; 95% CI 3.7–115.4); however, the average PCT levels did not differ significantly between the three AP severity groups (Fig 4C).

Calcium levels below 2 mmol/L were associated with severe AP (OR 5.2; 95% CI 1.5–17.7); however, average calcium levels did not differ significantly between the three AP severity groups (Fig 4D).

Triglyceride (Tg) levels above 40 mmol/L were associated with severe AP (OR 4.1; 95% CI 1.3–13.6); however, average Tg levels did not differ significantly between the three AP severity groups (Fig 4E).

An association between discrete glucose levels and AP severity was not observed; however, the average glucose levels differed significantly between mild vs. moderate and mild vs. severe AP cases (Fig 4F).

Haematocrit values, thrombocyte counts and serum levels of amylase, lipase, sodium (Na), potassium (K), lactate dehydrogenase (LDH), cholesterol, (S5A–S5H Fig), glutamic oxaloacetic transaminase (SGOT), glutamic pyruvic transaminase (SGPT), alkaline phosphatase (ALP), gamma-glutamyl transferase (GGT), direct bilirubin (diBi), creatinine and blood urea nitrogen (BUN) (S6A–S6G Fig) showed no significant changes with severe AP regardless of the method of analysis.

### Complications

**Fig 5:**
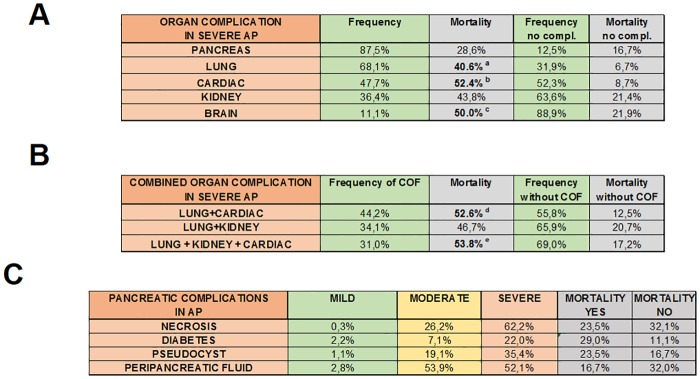
Frequency of organ failure and mortality in AP. **A.** Frequency of individual organ failure (pancreas, lung, cardiac, kidney and brain) and mortality in severe AP. **B.** Frequency of combined organ failure and mortality in severe AP. **C.** Frequency of pancreatic complications and mortality in AP. Mortality was only calculated in severe AP. **a:** p = 0.020 (Fisher’s exact test); **b:** p = 0.002 (Chi-square test); **c:** p = 0.043 (Fisher’s exact test); **d:** p = 0.003 (Chi-square test); **e:** p = 0.030 (Fisher’s exact test).

The most common organ complication in severe AP was of pancreatic origin (87.5%), followed by lung (68.1%), cardiac (47.7%), kidney (36.4%) and brain (11.1%) injury (Fig 5). Mortality in severe AP without respiratory failure was 6.7%, whereas it increased to 50% if lung injury persisted for more than 24 hours. Mortality in severe AP with no cardiac failure was 8.7%, while it was elevated to 57.1% if cardiac failure lasted for more than 24 hours. The lack of kidney failure did not decrease mortality in severe AP; however, if kidney failure persisted for more than 24 hours, the mortality rate was 50% (Fig 5). The rate of pancreatic local complications increased from mild to severe AP. The most common local complication was peripancreatic fluid (mild AP: 2.8%; moderate AP: 53.9%; severe AP: 52.1%). Importantly, local complications during AP had no effect on the risk of mortality (Fig 5).

### Conservative Therapy

**Fig 6:**
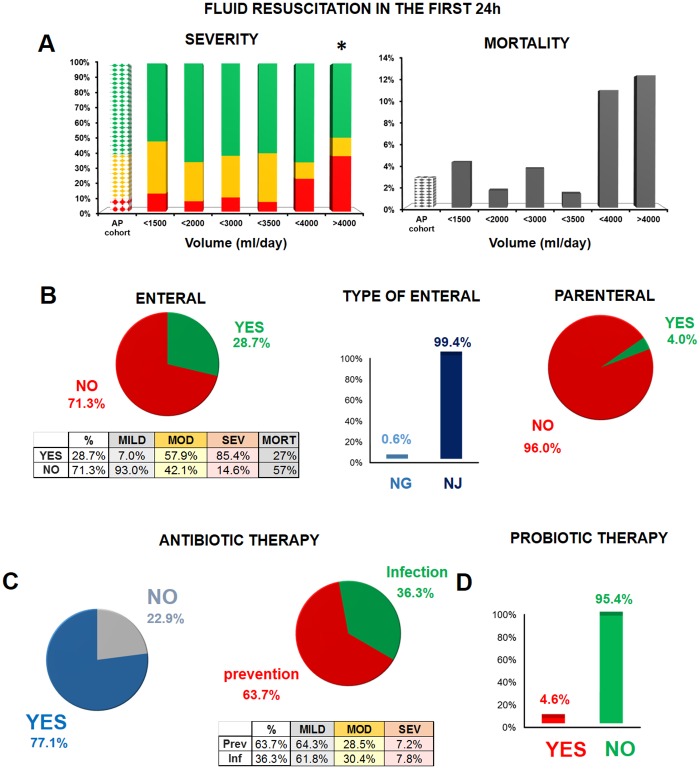
Conservative therapy in AP. **A.** Effect of fluid resuscitation on severity and mortality in the first 24 hours. The first dotted column represents the AP severity groups and mortality for each group in the entire cohort. Green: mild AP; yellow: moderate AP; red: severe AP; *: p = 0.030 (Fisher’s exact test on severity) versus the cohort (n = 8–185). A polynomial regression curve was fitted to demonstrate the mortality trend (n = 8–185). **B.** Enteral and parenteral feeding in AP. Mortality is shown for the severe AP group. NG: nasogastric feeding; NJ: nasojejunal feeding. **C.** Antibiotic therapy and its indications in AP. Table shows the indications for antibiotic therapy in the three severity groups. **D.** Probiotic therapy in AP.

Statistical analyses of fluid resuscitation practices in the first 24 hours showed that both severity and mortality are affected by the amount of fluid administered. The optimal fluid amount was between 1500 and 3500 mL (Fig 6A).

Enteral feeding was employed in 28.7% of the patients. In 99.4% of the cases, it was via nasojejunal tube (Fig 6B), while a nasogastric tube was used in one case. The majority (85.4%) of the patients with severe AP received enteral feeding. In severe AP, the mortality rate rose from 27% to 57% when enteral feeding was not administered (Fig 6B). Parenteral feeding was only employed in 4% of the cases.

The practice of antibiotic therapy was widespread in our cohort (77.1% of the cases) (Fig 6C). In two-thirds of the cases, the indication was for the prevention of infectious complications. There were no relevant differences in mortality or severity between patients who received antibiotics for prevention and those who were treated with antibiotics for infection. Since preventive antibiotics did not appear to be beneficial in our cohort, we can agree with the notion that use of preventive antibiotics is not beneficial (Fig 6C). The most common therapy was a combination of a cephalosporin and metronidazole (42.5%), whereas imipenem was only initiated in 5.5% of the patients. Antibiotic therapy was changed from cephalosporin/metronidazole or other combinations to imipenem in 6.9% of the cases. Although probiotics and proton pump inhibitors (PPIs) are not recommended by the guidelines, 4.6% of the patients received probiotics and 64.5% were administered PPIs (Fig 6D).

### Endoscopic Therapy

**Fig 7:**
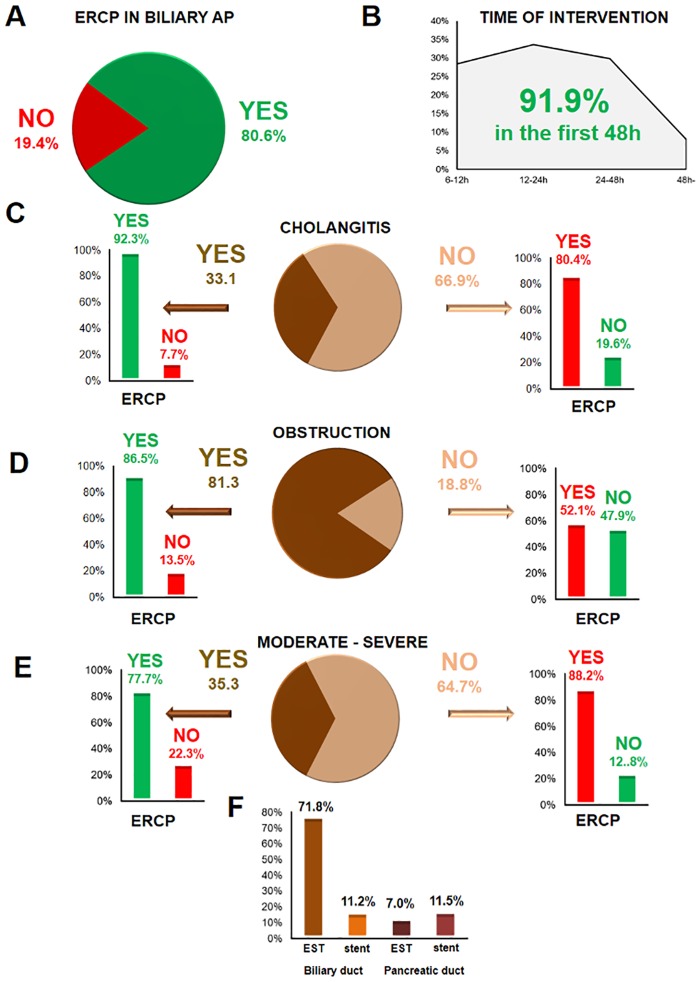
Endoscopic therapy in AP. **A.** ERCP in biliary pancreatitis. **B.** Time of intervention after hospital admission. **C–E.** Indication for ERCP in the presence of cholangitis, obstruction, and moderate or severe AP. Unjustified ERCP (mild AP with neither cholangitis nor obstruction) was only performed in 3.8% of the cases. **F.** Interventions during ERCP. EST: endoscopic sphincterotomy.

The cause of AP was biliary in 43.8% of the cases. In 18.8% of the cases, the diagnosis was based on laboratory alterations only (predicted AP) with no sign of obstruction. In 80.6% of the patients with biliary aetiology, ERCP was performed (Fig 7A). In 91.9% of the cases, the endoscopic intervention was carried out within the first 48 hours after the diagnosis of AP (Fig 7B). In 33.1% of the patients, cholangitis was present (Fig 7C), 81.3% had obstruction (Fig 7D), and 35.3% had severe or moderate AP (Fig 7E). Most ERCP procedures were conducted when cholangitis was present, whereas the fewest were performed when obstruction was not visible. Only 3.8% of the 212 ERCPs were unjustified (mild AP with neither cholangitis nor obstruction). Endoscopic biliary sphincterotomy was carried out in 71.8% of the cases, while endoscopic pancreatic sphincterotomy was conducted in only 7%. A biliary stent was placed in 11.2% of the cases, and 11.5% of the patients received a pancreatic stent.

### Interventions

**Fig 8:**
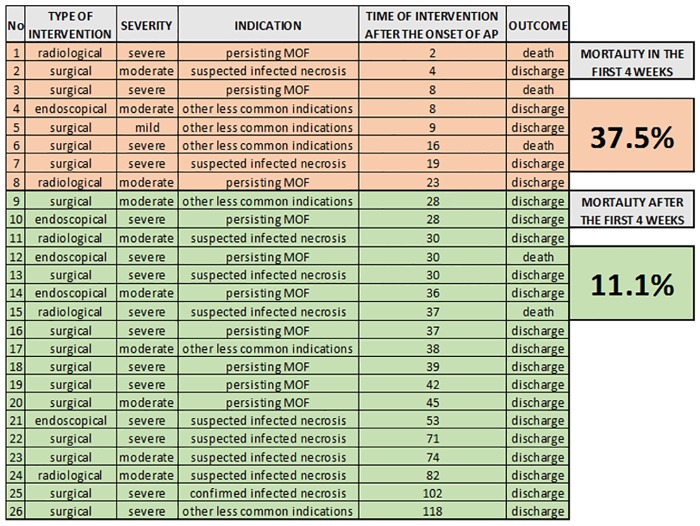
Type, indication and outcome of interventions in AP. Data show the mortality differences between early and late interventions in AP.

Interventions were performed in 26 cases (4.3%) in our cohort, of which 62% were surgical operations, 19% endoscopic procedures and 19% radiological interventions (Fig 8).

## Discussion

Our study contains among the largest cohorts of any publication on prospectively collected clinical data in AP to date. With regard to epidemiology, our study confirmed findings from other cohorts showing that AP in women is more likely related to gallstones, alcohol-related pancreatitis is more common in men [13, 14], and age is a risk factor for AP [15]. The role of dietary factors in the risk for AP is unclear. In experimental animal models, fatty acids, alcohol and fatty acid ethyl esters can cause AP; therefore, we separated “alcohol plus high-fat diet” as a distinct etiological group from alcohol-induced AP [16–18]. However, no difference was found in the severity of AP between cases associated with alcohol plus high-fat diet versus alcohol only.

Cigarette smoking has been linked to AP [19, 20]; however, the exact mechanism is unknown. Smoking also heightens the risk for recurrent AP [21]. In our study, we observed that smoking was connected with greater mortality in severe AP.

Hypertriglyceridaemia was an etiological factor of AP in our cohort, as described for other cohorts [22–25]. However, it is always a question whether elevation of serum triglyceride level is the cause of AP or merely a consequence of alcohol consumption and/or the hypertriglyceridaemia is just a coincidence. A rise in a serum triglyceride level above 11.3 mmol/L in a patient with AP is considered diagnostic, whereas above 5.6 mmol/L considered suspicion for hypertriglyceridemia-induced AP [23]. In our study, an increase in serum triglycerides above 5.6 mmol/L was considered as causative whenever no other obvious aetiology of AP was apparent. Experimental data have suggested that hypertriglyceridaemia can heighten the risk of severe AP [26]; however, the role of hypertriglyceridaemia in the risk for severe AP has been unclear so far [23]. One of the most important new observations of this study is that hypertriglyceridaemia is associated with severe AP. It has recently been shown that lipolysis of visceral adipocyte triglyceride converts mild AP to severe AP, independent of necrosis and inflammation [27]. Moreover, data suggest that the unsaturated fatty acids in the triglycerides used in intravenous lipids may contribute to the development of organ failure in AP [28].

Obesity has been shown to be associated with an elevated systemic inflammatory response and increased risk for systemic complications and mortality [29–32]. Although we could see a tendency between the rise in BMI and risk of severe AP, statistical analyses could not confirm the literature data.

With regard to laboratory parameters, a WBC count above 23,000/μL, CRP levels above 200 mg/L, PCT levels above 10 U/L, calcium levels below 2 mmol/L and triglyceride levels above 40 mmol/L were all associated with severe AP. Average glucose levels differed significantly between the three AP severity groups. Although others have reported that a haematocrit value above 44% and a rise in BUN are associated with persistent organ failure [33], our results could not confirm these findings.

Our study supports the recommendations in the IAP/APA guidelines. The first 24 hours were found to be the “golden hours” in AP [34, 35]. Recommendations D9–10 highlight the importance of fluid resuscitation at admission. In our cohort, fluid volume below 1500 mL or above 3500 mL raised the risk of mortality and AP severity. In the current study, 71.7% of the patients received the recommended amount of fluid. In this group, the severity rate was only 7.8%, whereas it increased to 14.1% in patients who were given less than 1500 mL or more than 3500 mL. Moreover, the mortality rate was also elevated from 2.3% to 5.1%. If we consider that around 7,000 new AP cases are diagnosed in Hungary in a year, hypothetically, we would prevent 124 patients from developing severe AP if fluid therapy were administered according to the IAP/APA guidelines. This would result in cost savings of HUF 200 million (about 672,000 euros) and could save 57 lives in a country of 10 million people. Projection of these calculations to the entire Eastern European region, with a population of about 200 million people, indicates that implementation of the EBM guidelines could save over a thousand lives.

Although there is no clear evidence for the efficacy of preventive antibiotic therapy [36, 37], recent meta-analyses of six randomized clinical trials point to reduced mortality and lower incidence of infected pancreas necrosis [38]. The recent Japanese guidelines suggest that in severe AP prophylactic antibiotic therapy may improve prognosis if started in the early phases of AP [39]. Recommendations F17–18 in the IAP/APA guidelines do not endorse prophylactic antibiotic therapy. Our data have provided further support for the latter recommendation since there was no difference seen in severity and mortality between groups who received preventive and therapeutic antibiotic treatments. Meta-analyses have shown that probiotics have neither beneficial nor adverse effects in the outcome of AP [40]. Our doctors generally followed recommendation F19 in the IAP/APA guidelines.

Recommendations on nutrition (G21) clearly suggest the importance of enteral feeding in severe and predicted severe AP. Recent meta-analyses have revealed that gut barrier dysfunction is present in three out of five patients in AP [41], thus raising the question whether enteral feeding is recommended in all types of AP. Although enteral feeding via a nasogastric tube is efficacious in severe AP [42–46], it is not among the preferred therapeutic tools in Hungary. In our cohort, we found that lack of enteral feeding was associated with mortality. Since the BISAP score has limitations [15, 47], our observation also highlights the need for a severity index in AP that could be employed at admission [48].

There is a lack of consensus on the role of ERCP in acute biliary AP [49]. It has been generally agreed that early ERCP should be performed in biliary AP with cholangitis [6, 50–52]; however, there is only limited evidence available whether early ERCP should be conducted when only duct obstruction is present [50, 53]. The strongest controversy concerns the predicted severe biliary pancreatitis without cholangitis and obstruction [49]. The IAP/APA guidelines suggest that ERCP is most probably not indicated in the latter case [6]. In our cohort, most of the endoscopists followed the guidelines when cholangitis was observed; however, they used a defensive attitude in cases of obstruction and predicted severe AP.

An unexpectedly large number (30.7%) of the interventions were performed within the first four weeks after the onset of AP. Although the clinical necessity (which was not investigated in this cohort) could have indicated the procedure, our cohort suggests a lack of awareness of recommendation J31 on the necessity of delaying the intervention for at least four weeks.

In summary, a number of important determinants and associations of severity and mortality in AP were identified in our large, prospectively collected, nationwide cohort. The results also highlight that the evidence-based IAP/APA guidelines should be followed strictly to improve outcomes in AP.

## Supporting Information

S1 Fig Distribution of participating centres. (TIF)

S2 Fig Prospectively collected parameters. 86 different parameters were collected. Overall, 77% of the data requested were provided by the investigators. Only four parameters (amount of smoking, amount of alcohol, Glasgow coma score and breath rate) were not analysed due to limited data.
(TIF)

S3 Fig Mortality peaks during AP. (TIF)

S4 Fig Lung complications at admission. Investigators ordered tests for pleural fluid or lung infiltration by abdominal US in 74.3% of the cases, by chest X-ray in 37.7% and by thoracic CT in 6.5%. The most positive results were found by thoracic CT (75.6%) followed by X-ray (26.6%) and abdominal US (6.7%). Severity and mortality data were analysed in groups with and without pleural complications. Data suggest that doctors are more likely to test for lung complications when severe AP is predicted.
(TIF)

S5 Fig Laboratory parameters. On the left panel of graphs laboratory parameters were analysed by distinct values, grouped in ranges. The first dotted column represents the AP severity groups of the entire cohort. In the right-hand panel of graphs the average laboratory parameters were compared in the three AP severity groups. Here, we used the Kruskal-Wallis test to analyse the significance level and Mann-Whitney U test with Bonferroni correction to compare the pairs of groups under examination. Green, mild AP, yellow, moderate AP, red, severe AP. **A**, Amylase (n = 64–165). **B**, Lipase (n = 12–130). **C**, Sodium (Na, n = 15–113). **D**, Potassium (n = 26–113). **E**, Lactate dehydrogenase (LDH, n = 32–43). **F**, Cholesterol (n = 15–59). **G**, Hematocrit (n = 9–95). **H**, Thrombocyte count (n = 24–116).
(TIF)

S6 Fig Laboratory parameters. For description of statistical analyses see S5 Fig. **A,** Glutamic oxaloacetic transaminase (SGOT, n = 19–44). **B**, Glutamic pyruvic transaminase (SGPT, n = 46–88), **C**, Alkaline phosphatase (ALP, n = 9–165), **D**, Gamma-glutamyl transferase (GGT, n = 31–109). **E,** Direct bilirubin (diBi, n = 11–31). **F**, Creatinine (n = 6–230), **G**, Blood urea nitrogen (BUN) (n = 59–226).
(TIF)

S7 Fig Summary of the cohorts. (TIF)
