# Supplementary figures and images for: Prospective, Multicentre, Nationwide Clinical Data from 600 Cases of Acute Pancreatitis
Source: PLoS One. 2016 Oct 31;11(10):e0165309. doi: 10.1371/journal.pone.0165309 (PMC5087847; doi:10.1371/journal.pone.0165309)

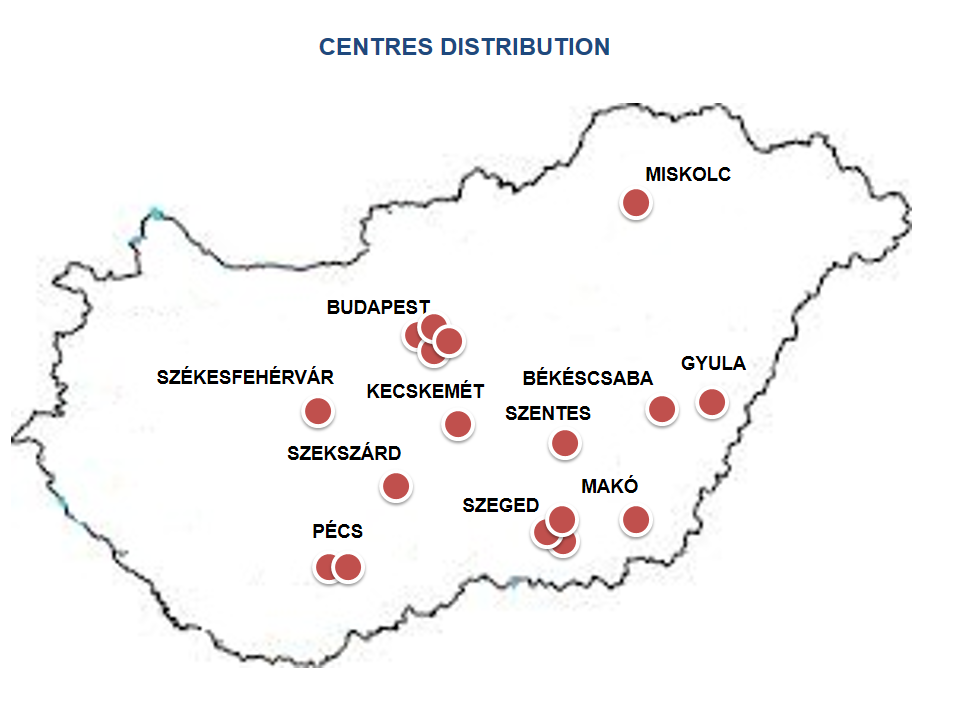

Supplement: S1 Fig — (TIF) [file pone.0165309.s001.tif]

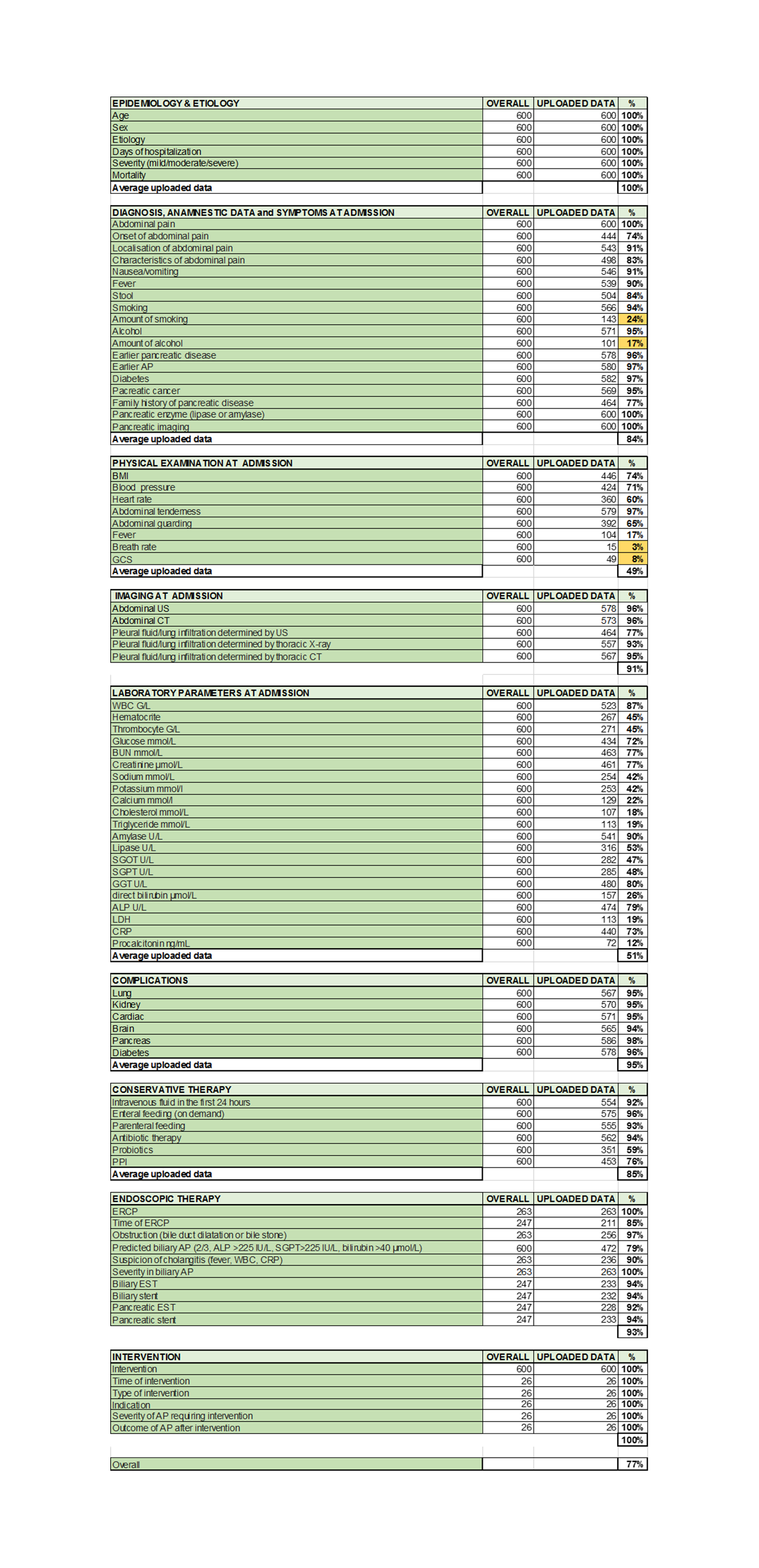

Supplement: S2 Fig — 86 different parameters were collected. Overall, 77% of the data requested were provided by the investigators. Only four parameters (amount of smoking, amount of alcohol, Glasgow coma score and breath rate) were not analysed due to limited data. (TIF) [file pone.0165309.s002.tif]

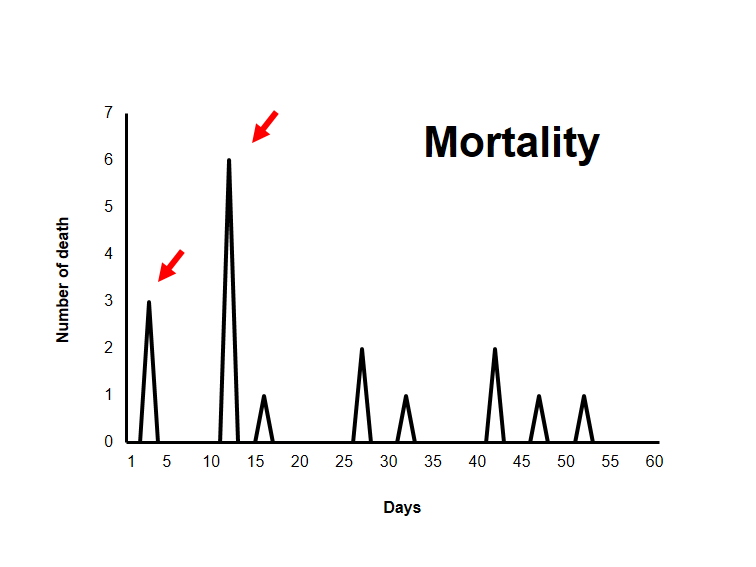

Supplement: S3 Fig — (TIF) [file pone.0165309.s003.tif]

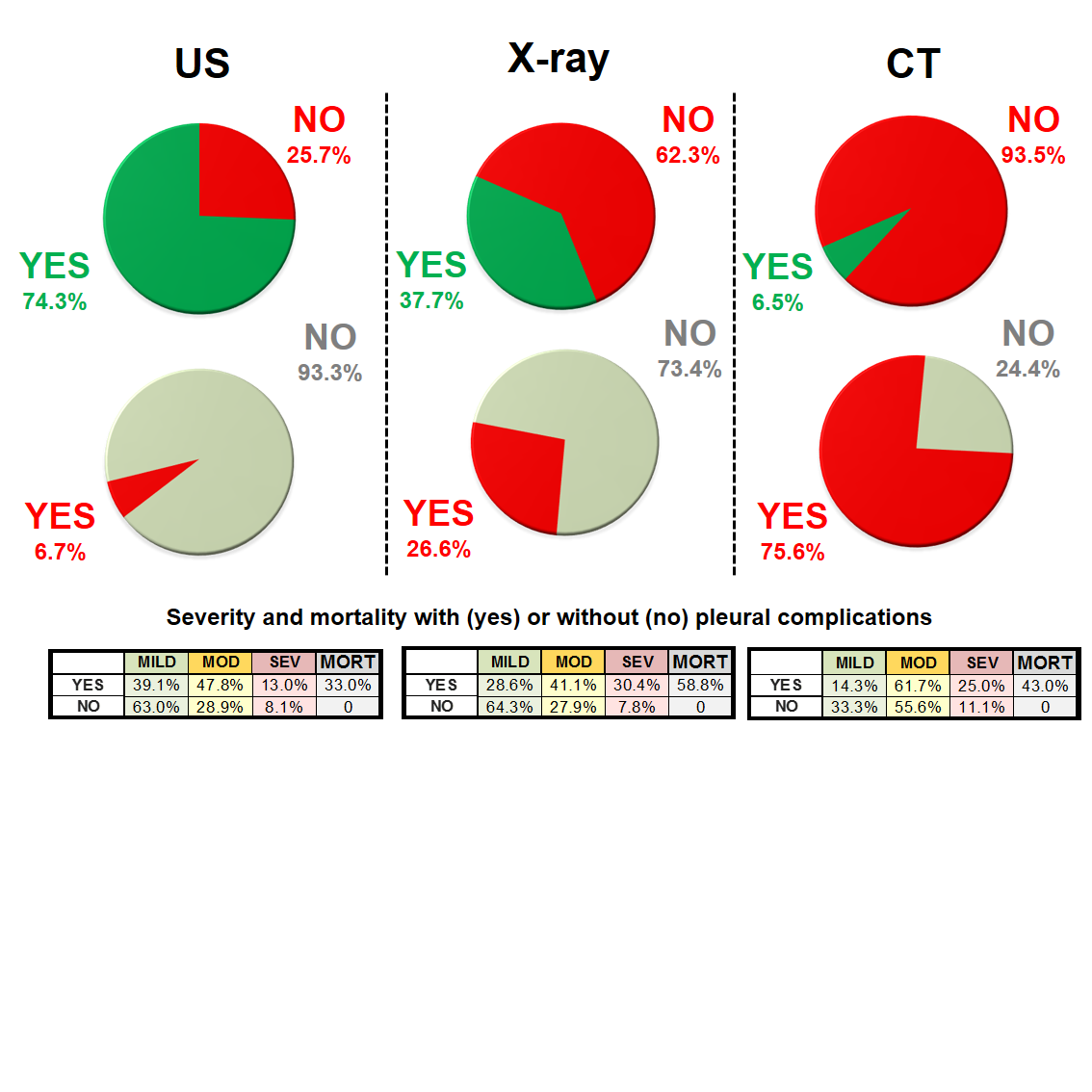

Supplement: S4 Fig — Investigators ordered tests for pleural fluid or lung infiltration by abdominal US in 74.3% of the cases, by chest X-ray in 37.7% and by thoracic CT in 6.5%. The most positive results were found by thoracic CT (75.6%) followed by X-ray (26.6%) and abdominal US (6.7%). Severity and mortality data were analysed in groups with and without pleural complications. Data suggest that doctors are more likely to test for lung complications when severe AP is predicted. (TIF) [file pone.0165309.s004.tif]

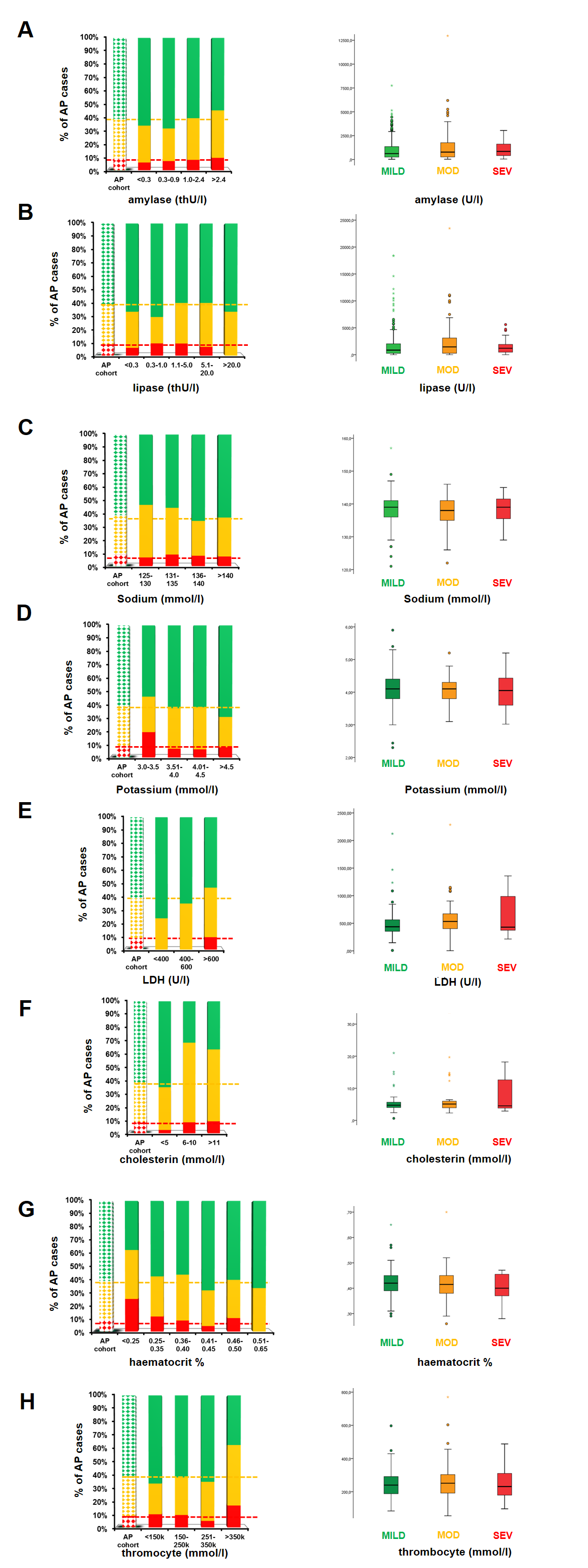

Supplement: S5 Fig — On the left panel of graphs laboratory parameters were analysed by distinct values, grouped in ranges. The first dotted column represents the AP severity groups of the entire cohort. In the right-hand panel of graphs the average laboratory parameters were compared in the three AP severity groups. Here, we used the Kruskal-Wallis test to analyse the significance level and Mann-Whitney U test with Bonferroni correction to compare the pairs of groups under examination. Green, mild AP, yellow, moderate AP, red, severe AP. A, Amylase (n = 64–165). B, Lipase (n = 12–130). C, Sodium (Na, n = 15–113). D, Potassium (n = 26–113). E, Lactate dehydrogenase (LDH, n = 32–43). F, Cholesterol (n = 15–59). G, Hematocrit (n = 9–95). H, Thrombocyte count (n = 24–116). (TIF) [file pone.0165309.s005.tif]

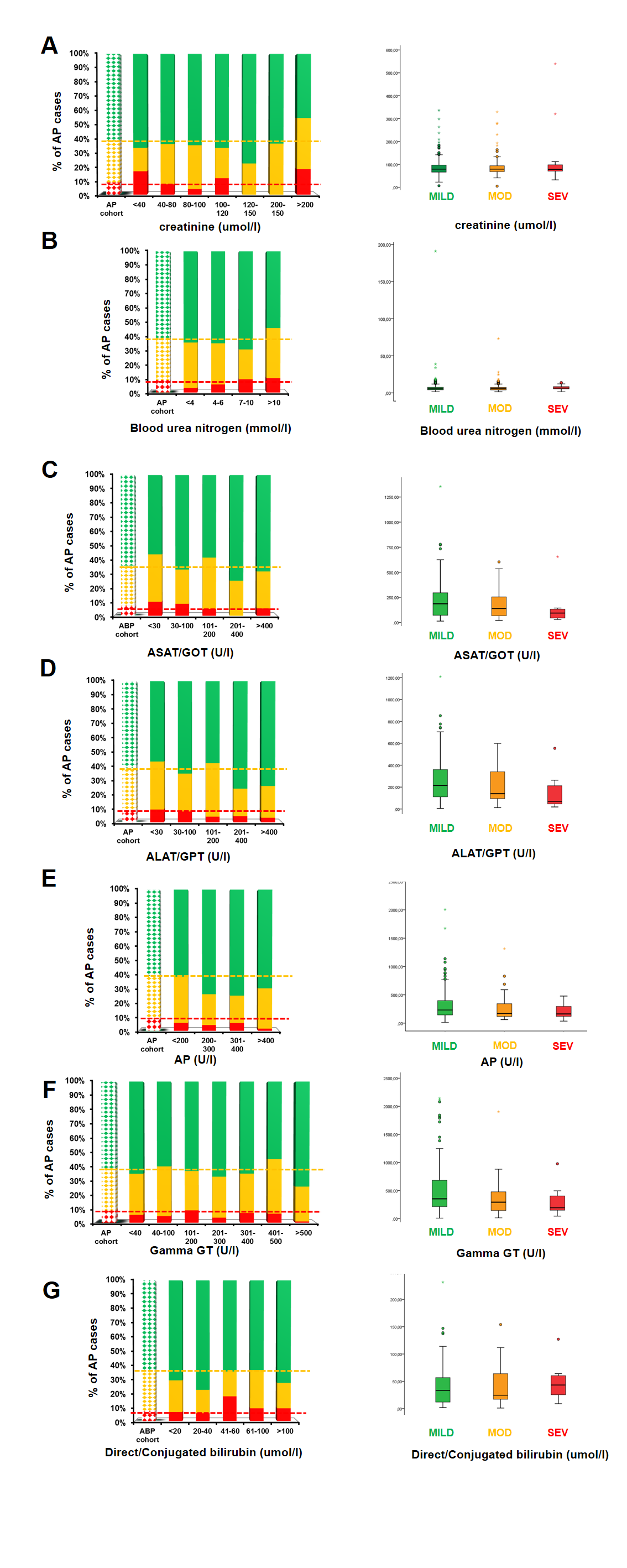

Supplement: S6 Fig — For description of statistical analyses see S5 Fig. A, Glutamic oxaloacetic transaminase (SGOT, n = 19–44). B, Glutamic pyruvic transaminase (SGPT, n = 46–88), C, Alkaline phosphatase (ALP, n = 9–165), D, Gamma-glutamyl transferase (GGT, n = 31–109). E, Direct bilirubin (diBi, n = 11–31). F, Creatinine (n = 6–230), G, Blood urea nitrogen (BUN) (n = 59–226). (TIF) [file pone.0165309.s006.tif]

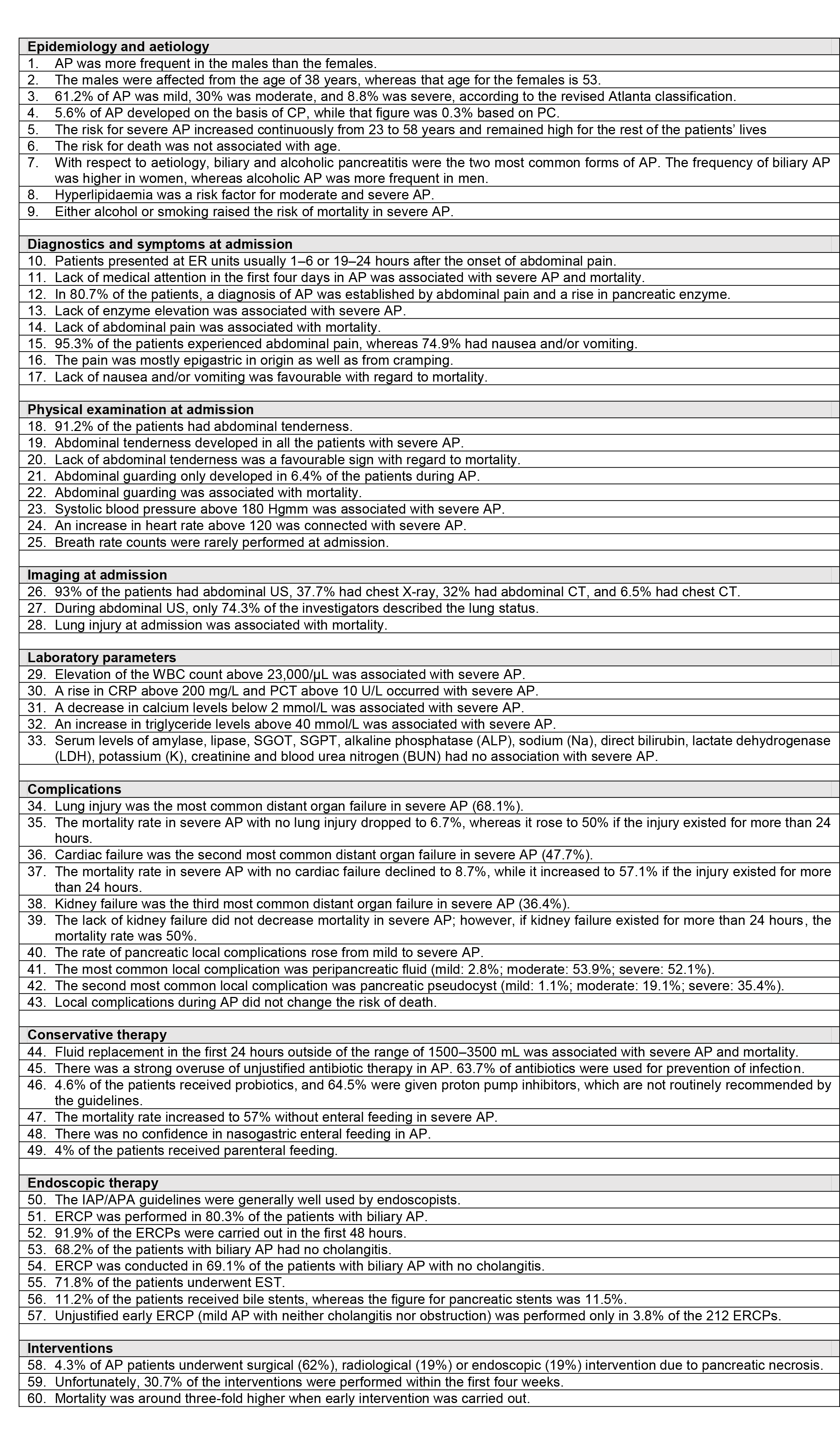

Supplement: S7 Fig — (TIF) [file pone.0165309.s007.tif]
